# Towards improving aspect-oriented software reusability estimation

**DOI:** 10.1038/s41598-024-62995-z

**Published:** 2024-06-08

**Authors:** Aws A. Magableh, Hana’a Bani Ata, Ahmad A. Saifan, Adnan Rawashdeh

**Affiliations:** 1 Department of Information Systems, Faculty of Computer Sciences and Information Technology, Yarmouk University, Irbid, 21163 Jordan; 2 Departments of Software Engineering, Prince Sultan University, Riyadh, Saudi Arabia

**Keywords:** Aspects, Aspect-oriented, AO, Quality attribute, Metrics, Reuse, Software reusability, Information technology, Software

## Abstract

Nowadays, large numbers of organizations may opt for Aspect-Oriented Programming (AOP), which is an enhancement to Object-Oriented Programming (OOP). This is due to the addition of a number of concepts that have assisted in the production of more flexible and reusable components. One of the most important elements added by AOP is software reuse, which is based on reusability attributes. These attributes indicate the possibility of reusing one or more components in the development of a new system. It is one of the most essential attributes to evaluate the quality of a system’s components. Thus far, little attention has been paid to the process of measuring AOP reusability, and it has not yet been standardized. The objective of the current study is to come up with a reasonable measurement for AOP software reuse, which is simultaneously a significant topic for researchers while offering several advantages for organizations. Although numerous models have been built to estimate the reusability of software, most of them are not dedicated to Aspect-Oriented Software (AOS). In this study, a model has been designed for AOS reusability estimation and measurement based on a new equation depending on five attributes that have a range of positive and negative impacts on AOS reusability. Three of those attributes, namely coupling, cohesion, and design size, have been included in previous studies. This study proposes complexity and generality as two new attributes to be considered. Each of these attributes was measured based on the metrics also proposed in this study. A new equation to calculate AOS reusability was constructed based on the most important reusability attributes and metrics. Seven aspect projects were employed as a case study to apply the proposed equation. After the proposed equation was applied to the selected projects, we obtained new values of reusability to compare with the values that resulted from applying the previous equation. The fact that new values emerged indicates that the proposed reusability metrics and attributes had a significant effect.

## Introduction

Aspect-oriented programming (AOP) significantly contributes to software reusability by addressing one of the key challenges in software development: the effective management of cross-cutting concerns. By modularizing these concerns separately from the core application logic, AOP facilitates the creation of reusable components that can be easily applied across different projects or modules^1^. This modularization promotes code reuse by enabling developers to encapsulate common functionalities, such as logging, caching, or security, into aspects that can be easily integrated into various parts of the codebase. Consequently, AOP enhances the overall reusability of software components, leading to more efficient development processes and the creation of more maintainable and scalable software systems.

The industrial sector's rapid expansion owes much to the continual evolution of software, now integral to most organizations^2,3^. Consequently, the demand for lines of code escalates, leading to increased costs^4,5^. To mitigate these challenges, organizations employ software reuse strategies, aiming to streamline development processes, cut costs by up to 20%, and enhance efficiency^6^. Scholars such as^1,7–9^, and^4^ have contributed various definitions of software reuse, all converging on a central concept: leveraging pre-existing components for constructing new software, thereby maximizing efficiency and reducing redundancy.

Object-Oriented Programming (OOP) emerged to support the software reuse principle in building software. The OOP paradigm is used to implement the software by structuring the system using simple and reusable components like modules, concepts called classes and objects, in addition to many other fundamental OO concepts and principles, including encapsulation, inheritance, abstraction, and polymorphism^10^. Thanks to these features, dealing with systems became simpler, but as the size of these systems grew over time^3^, the development process became more difficult and involved high dependency. In addition, coupling appeared between the objects, necessitating the search for a solution that addresses these issues and reduces risks^11^. More recently, the Aspect-Oriented Programming (AOP) paradigm has emerged as an extension of the OOP paradigm to improve the development process and to produce more reusable software^12^. AOP is based on the modularization principle, which aims to decompose the system into separation concerns called “crosscutting modules” or “aspects” with less dependency or coupling because all crosscutting concepts are collected into the same module to reduce the relationships as much as possible and to make the software more maintainable^13^.

To adapt to the paradigm shift in development and the growing reliance on software, while simultaneously managing costs effectively, prioritizing the concept of reusability throughout the Software Development Life Cycle (SDLC) emerged as the optimal solution. This approach has spurred numerous studies on reusability^5,14^. The benefits of reuse extend to various critical software attributes, including heightened productivity, enhanced quality, and bolstered reliability^15,16^. Assessing the reusability aspect involves the use of several metrics aimed at gauging different facets of software. Through the thorough examination, analysis, and quantification of these metrics, decision-makers can make more informed choices throughout the reuse process^11^.

The reusability of software is a major approach that companies have an interest in. Software reusability has been defined as “the ability of a software component to be used in different contexts repeatedly”^11^ and “the degree to which a thing can be reused”^17^.^18^ defined software reuse as “the depth to which a module can be reused again with very little or no modification”, characterized it as “a method in which a part of software design can be reused by adding additional functionalities onto the existing one with little or no modification”. Due to the importance of software reusability, the most popular metrics and attributes have been discussed during this study. The correlations between these attributes and their effects on software quality and reusability have been investigated using the models that are based on them. The study also seeks to determine which ones are particularly relevant to the AOS and study them in depth to discover and summarize the metrics and attributes that are directly related to the reusability of AOS. Furthermore, we investigated the previously existing models to properly evaluate the approaches, and finally designed a model to achieve an estimation of the AOS reusability.

In navigating the landscape of reusable components, organizations and developers often grapple with identifying the most suitable ones for their systems. The challenge arises from the need for substantial modifications to adapt available components to their specific requirements, thereby impeding the development process and inflating costs^19^. Developers seek a method to effectively evaluate the reusability of components, facilitating informed decision-making regarding the reuse process^20,21^. The absence of comprehensive models focusing on the reusability of components in the domain of AOS serves as the driving force behind this research. In contemporary software development, reusability holds significant importance, as starting from scratch entails substantial resource expenditure in terms of finances, manpower, equipment, and time.

Moreover, the Aspect-Oriented (AO) concept enriches numerous software properties and attributes, particularly when adhering to specialization and modularization principles, thereby strongly advocating for the concept of reuse and enhancing system reusability. Consequently, developers relying on Aspect-Oriented Systems (AOS) must gauge the reusability of AOS components available for integration into other systems to facilitate informed decision-making regarding reuse. This study delves into an exhaustive examination of all metrics and attributes impacting AOS reusability from diverse perspectives. It proposes a model based on the most significant metrics while also introducing novel metrics and attributes pertinent to AO software reusability. Through regression analysis conducted in SPSS, the study aims to pinpoint significant software metrics by scrutinizing the correlation coefficient between the dependent variable—AOS reusability—and independent variables encompassing both Object-Oriented (OO) and Aspect-Oriented (AO) metrics. This analysis allows us to explore the relationship between changes in the independent variables and variations in the dependent variable, offering insights into the software metrics that contribute most to AOS reusability. By understanding the strength and direction of these relationships, we can make informed decisions in software development and improve the overall reusability and efficiency of Aspect-Oriented Systems (AOS).

This research will attempt to address the following two significant questions:

- RQ1: To what extent can the research identify the reusability metrics and attributes that are in common between AO and OO software?
- RQ2: What are the special reusability metric attributes of AOS?
- RQ3: What are the possibilities for proposing an AO software reusability estimation equation?

The remainder of the article is organized as follows: Section “Background and literature review” explains the background and literature review. Section “Methodology and proposed approach” then shows the methodology followed throughout this research, while section “AOS reusability metrics and attributes” illustrates the results and evaluation. Finally, section “AOS reusability estimation” elaborates on conclusions and future work.

## Background and literature review

In this section, the researcher explains and adapts several basic concepts that need to be identified in order to understand the study’s key contributions with the aid of existing relevant research.

### OOP and AOP

There are many paradigms that have been used by developers to design systems, such as OOP and AOP, both of which are advantageous approaches to implementing software, giving it good properties and improving its quality.

**Figure 1:**
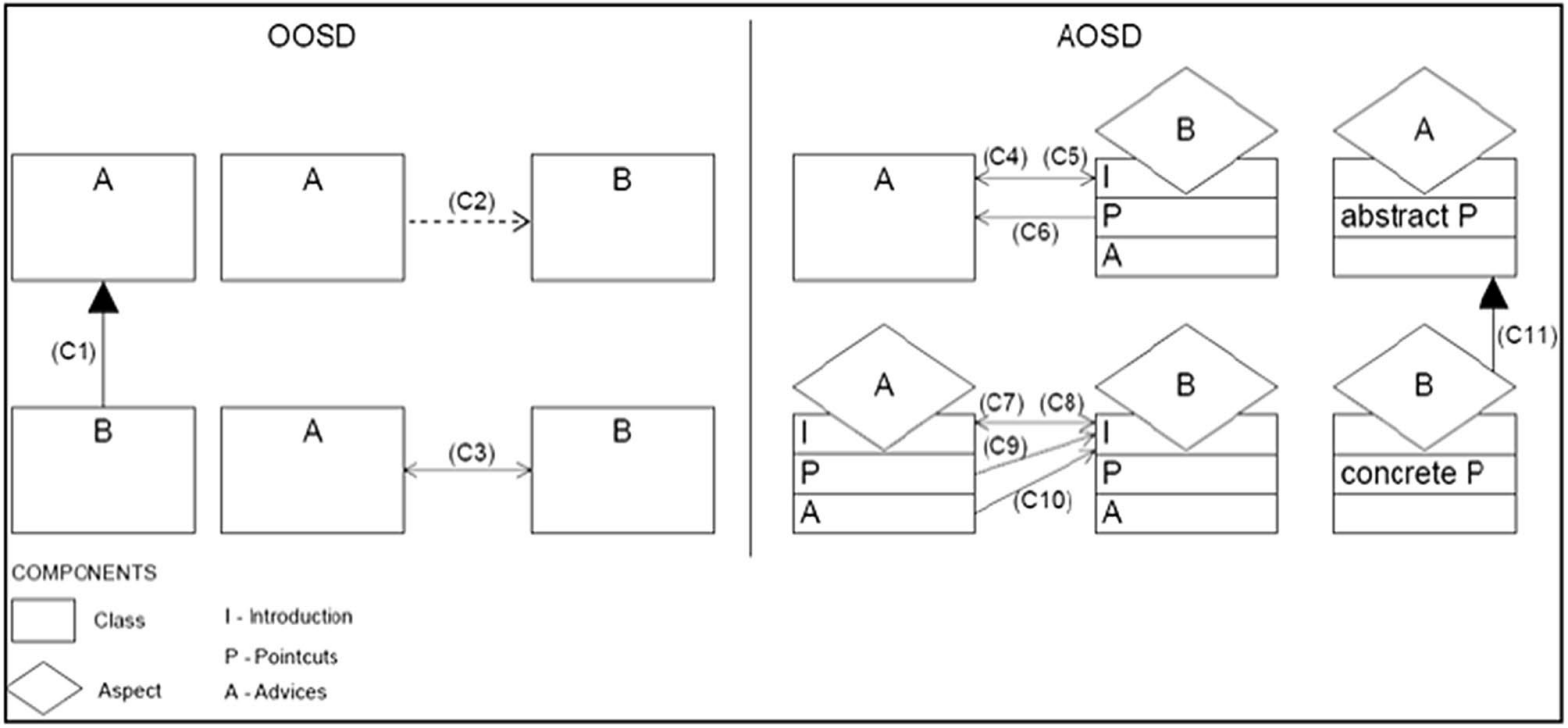
Implementation of OOP and AOP^24^.

OOP aims to support several significant attributes of software, like maintainability and reusability, which are achieved through features such as polymorphism, inheritance, encapsulation, aggregation, and abstraction. However, as the reliance on software grows, OOP becomes insufficient^22^, resulting in the emergence of new paradigms. AOP is an extension to OOP that has improved modularity, reusability, and maintainability^23^ by modularizing the crosscutting concerns into a single module called an ‘Aspect’. This in turn helps in dealing with software and makes it easier to reuse and maintain. As a result, AOP became the paradigm most commonly used by developers to implement systems. Figure 1 shows two simple OOP and AOP implementation examples that demonstrate the fundamental notions of each^24^.

AOP has become the center of attention since its emergence in 1996 at Xerox Palo Alto Research Centre (PARC). It appears to have added enhancements to OOP, by decreasing the complexity of the development process, and producing reusable components. The crosscutting concept is interested in gathering the components that have a common idea and functionality into one module or aspect to decrease the coupling degree in the system. This is achieved in accordance with the application of the encapsulation principle. These modules or aspects were established depending on many concepts that assist in their integration. Among the most important of these concepts is Join Point, which is used to invoke the methods or throw the exceptions. The advice concept helps the objects in the crossing process at a specific join point. The Pointcut concept is used to specify the place where advice should be given. AO systems have been implemented using many languages, such as the AspectJ language which provides good support, and the Generation of Dynamic Byte Code language^25^, which has also been widely used^26^. AspectJ is the most popular Java extension language that is used in AOP and provides protocol management, consistency checking, synchronization, and other services which give the application a more flexible design for easy failure detection, reuse, and maintenance^27^.

### Software quality metrics and models

Any system that is considered good software should be of high quality and should provide good benefits for different parties interested in it^21^, such as productivity for the organization’s management and efficient use for the user. Generally, depending on the methodology or model, many metrics and attributes could be considered to obtain a quality evaluation of any software. The most important and popular six metrics for OO software, as proposed by^28^ appear in Table 1^29^. They are used to evaluate several attributes that indicate the quality of the software^30^

**Table 1:** Chidamber and Kemerer metrics.

Metric	Description
Weighted Methods per Class (WMC)	It is measured by counting methods for each class
Depth of Inheritance Tree of a class (DIT)	It is measured by the length of the longest path at a module
Number of Children (NOC)	It is measured by counting the directly inherited classes for a parent class
Coupling Between Objects (CBO)	It is measured by counting the modules that have a relationship with the current module
Response for a Class (RFC)	It is measured by counting the methods in a class that respond to a message from outside class
Lack of Cohesion in Methods (LCOM)	It is measured by counting the null intersection methods set between classes

Based on these and other metrics, several models have been proposed to evaluate the quality of software^31,32^. The most important examples are listed as follows:

#### McCall’s quality model

**Figure 2:**
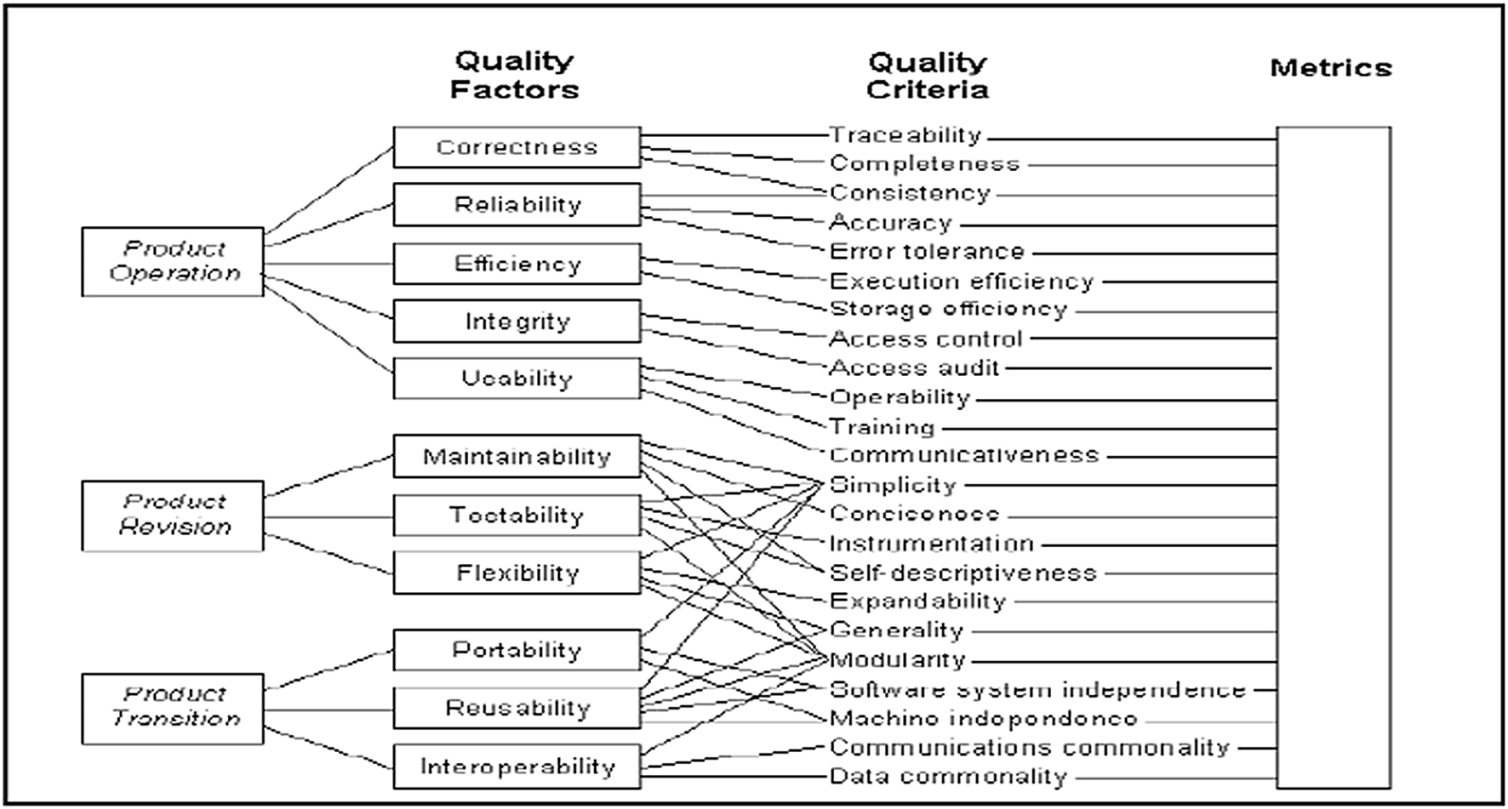
McCall’s quality model^34^.

McCall’s 1977 quality model, one of the first proposed models, takes into consideration three perspectives of the product, as shown in Fig. 2, and relates each one of them with the factors and attributes that affect it, pointing out that they are not directly measurable. Therefore, the quality criterion was used to measure them according to measurable metrics and to obtain a quantifiable quality of the product^33^.

#### Boehm’s quality model

**Figure 3:**
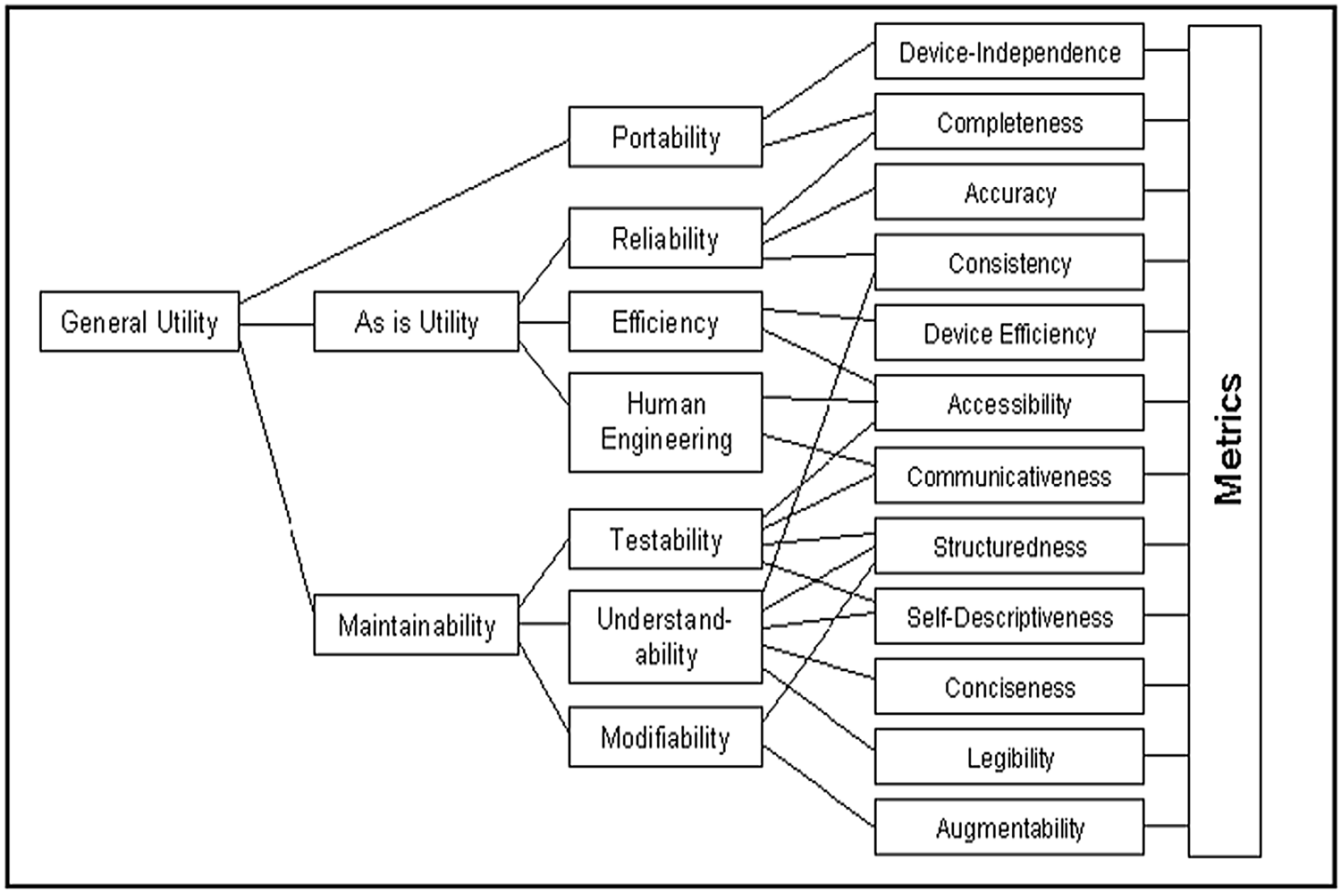
Boehm’s quality model^34^.

Proposed in 1978, Boehm’s quality model sought to improve on McCall's model by concerning itself with the general utility of the software, as shown in Fig. 3. From Boehm's point of view, the utility of the software includes three aspects, which are portability, as-is utility, and maintainability. If the software is able to achieve these, it is deemed to be of high quality. It depends on several aspects, such as non-measurable factors, software qualities that impact usefulness, and the metrics used to calculate them to evaluate software quality^33^.

#### Dromey’s quality model

**Figure 4:**
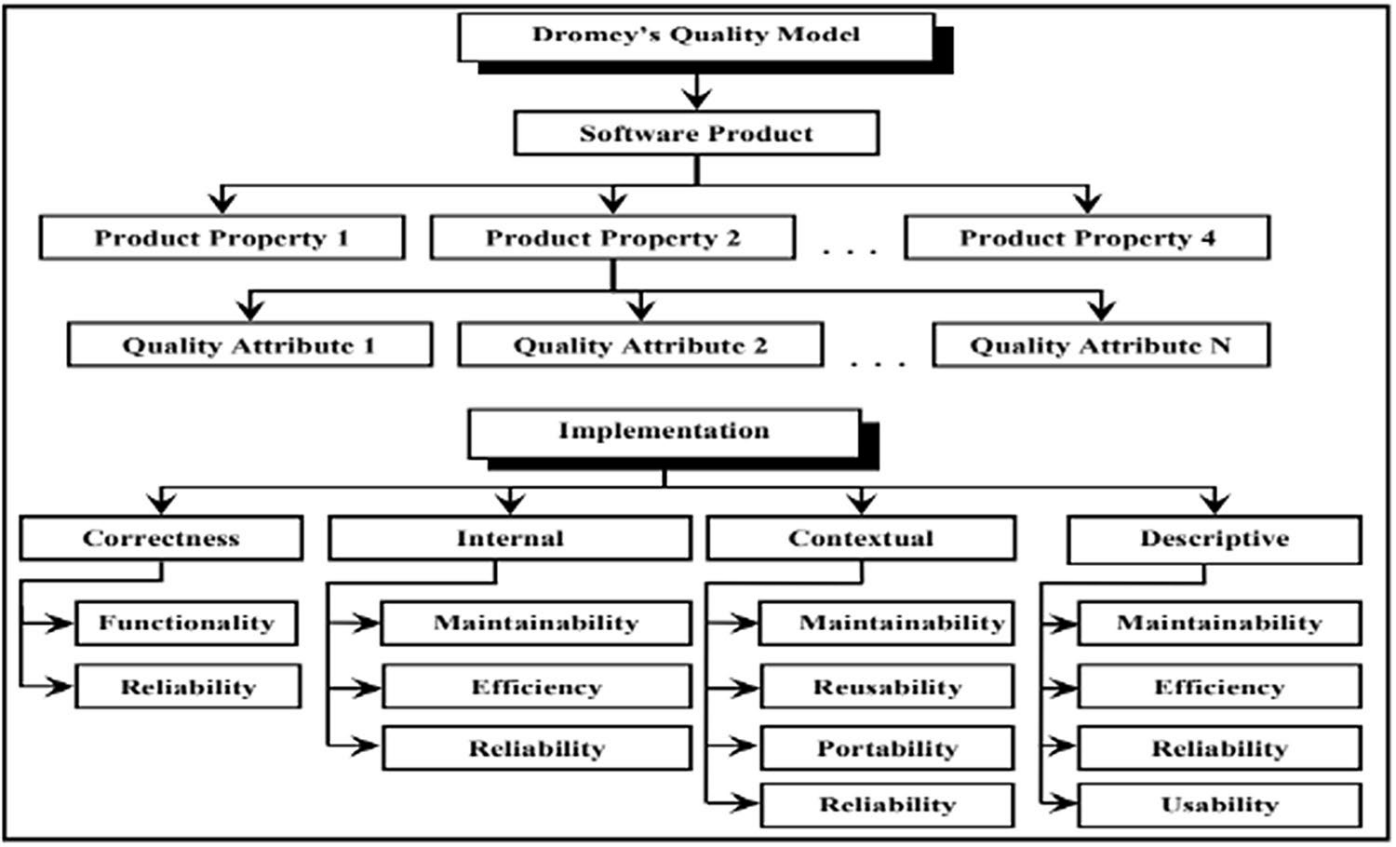
Dromey’s quality model and its contents^35^.

Dromey’s model was proposed in 1995 and highlights four properties of products, which are correctness, internal, contextual and descriptive, as shown in Fig. 4. Each model has several quality attributes that are used to finally determine the quality of the software^35^.

According to the quality models previously mentioned, there are many common quality factors and attributes, and they are all practically based on the same primary metrics at the calculation level to complete the evaluation process. As noted, Dromey's quality model has reusability as an important contextual attribute and there are other proposed models that have an interest in it, such as the Kazman Model (2003), and the Component-based Software Development Quality Model (2008)^32^.

In OOP or AOP, the measurement and evaluation of software quality metrics and attributes have faced many challenges and limitations, such as the field for which the system was built. A highly complex system is often more difficult to evaluate. Finding a good model or criteria to measure and evaluate this system is challenging because not all of the proposed evaluation models are good enough for all systems. Furthermore, there may be some difficulty in measuring some metrics because there isn’t a direct approach to obtain their values.

### Software reusability metrics and models

Software reuse is considered to be an improvement key in today’s software engineering and depends on the reusability attribute, which is used to evaluate the possibility of a component being reused. Due to their importance and relevance, there are many previous studies that have been generally concerned with software reuse and reusability at any SDLC level, such as^36^ which addresses the reusability of design patterns, class level, and package level to investigate which one is the most reusable. The Reusing Value Assessment Framework (RVAF) presented by^37^ is used to evaluate the overall cost of the reusing process. The research of^38^ presents a discussion of software reusability requirements, resulting in the proposal of a Requirements Reuse Model for Software Requirements Catalog (RRMSRC), which is based on proposed guidelines and activities to reuse the functional requirements. It is also based on a catalog of the requirements to make the reuse process simpler and more effective.

In^7^, the researchers used three methods (interviews, respondents, and qualitative analysis of the coding process) to identify the factors that affect the reusability of Open-Source Software (OSS). They ultimately named nine reusability factors (attributes) as: flexibility, maintainability, portability, scope coverage, stability, understandability, usage history, variability, and documentation. OOP was used to design the systems and make them more reusable. Many previous studies have focused on OO reusability, such as^39^. Researchers have also discussed the identification of different methods used by the developers to assess the reusability of OOS. The researcher further discussed many factors that affect it, for example, the programmer's experience, the development skills, the technology that has been used in the development process, testing availability, maintained guidelines of the software, the cost, and finally, the effort that is required to assess the reusability by the organization through the development process. Software reuse is applied in^5^ to develop a mobile application system based on domain analysis^40^ based their study on soft computing techniques to measure reusability. Many important attributes of reusability, and their metrics, are discussed by^41^. The researchers identified incurred reuse, adaptability, maintainability, external quality, availability, documentation, and complexity as being the most crucial attributes.

### AOS reusability metrics and models

AOP is an enhancement paradigm that appeared after OOP to ensure a greater quantity of high quality reusable software. AOP was established through many languages, the most important of which is AspectJ. Many previous studies were mainly concerned with AO reusability and its attributes, such as^24^ An assessment framework was proposed for Aspect-Oriented Software Development (AOSD) to obtain reusable software with less maintenance. This framework mainly depends on two levels; the first level relates to studying metrics that affect the reuse process, such as the separation of concerned metrics, coupling metrics, cohesion metrics, and size, and collecting meaningful data about them. This data was used in the second level, which is a quality model used to evaluate the maintainability and reusability of the software. In^14^ the author assessed the AOS reusability based on fuzzy logic methodology^30^, and they depended on many reusability attributes such as maintainability, understandability, adaptability, and modularity^42^. Additionally, in^43^analyzed and deeply discussed the quality characteristics of AO systems and the correlation between them. These scholars studied several important issues, such as the correlations between cohesion and (changeability and reusability), and the correlation between complexity and reusability. However, this study revealed the effects of two discovered attributes on reusability.^20^ studied AOSD, highlighting the main software quality parameters, which are reusability, maintainability, understandability, and testability. Moreover, the importance of reusability was explained in addition to the effect of maintenance on quality. The AOS reusability was studied at the package level^44^, who based their study on a framework concerned with many AOS reusability attributes such as maintainability, understandability, of adaptability, and modularity. Many metrics were defined for these attributes such as EC (Efferent Coupling) and PCohA (Package Cohesion Aspect).

There are quality models that are especially proposed to evaluate the quality attributes of AOS such as:

1. The first quality model for AOS, the Aspect–Oriented Software Quality Model (AOSQUAMO), was proposed by Kumar in 2009 as an extension of the ISO 9126-1 software quality model. As shown in Fig. 5, it has four sub-characteristics, which are modularity, code reducibility, complexity, and reusability, in addition to the original characteristics and sub-characteristics of the ISO 9126-1 model^27^.

**Figure 5:**
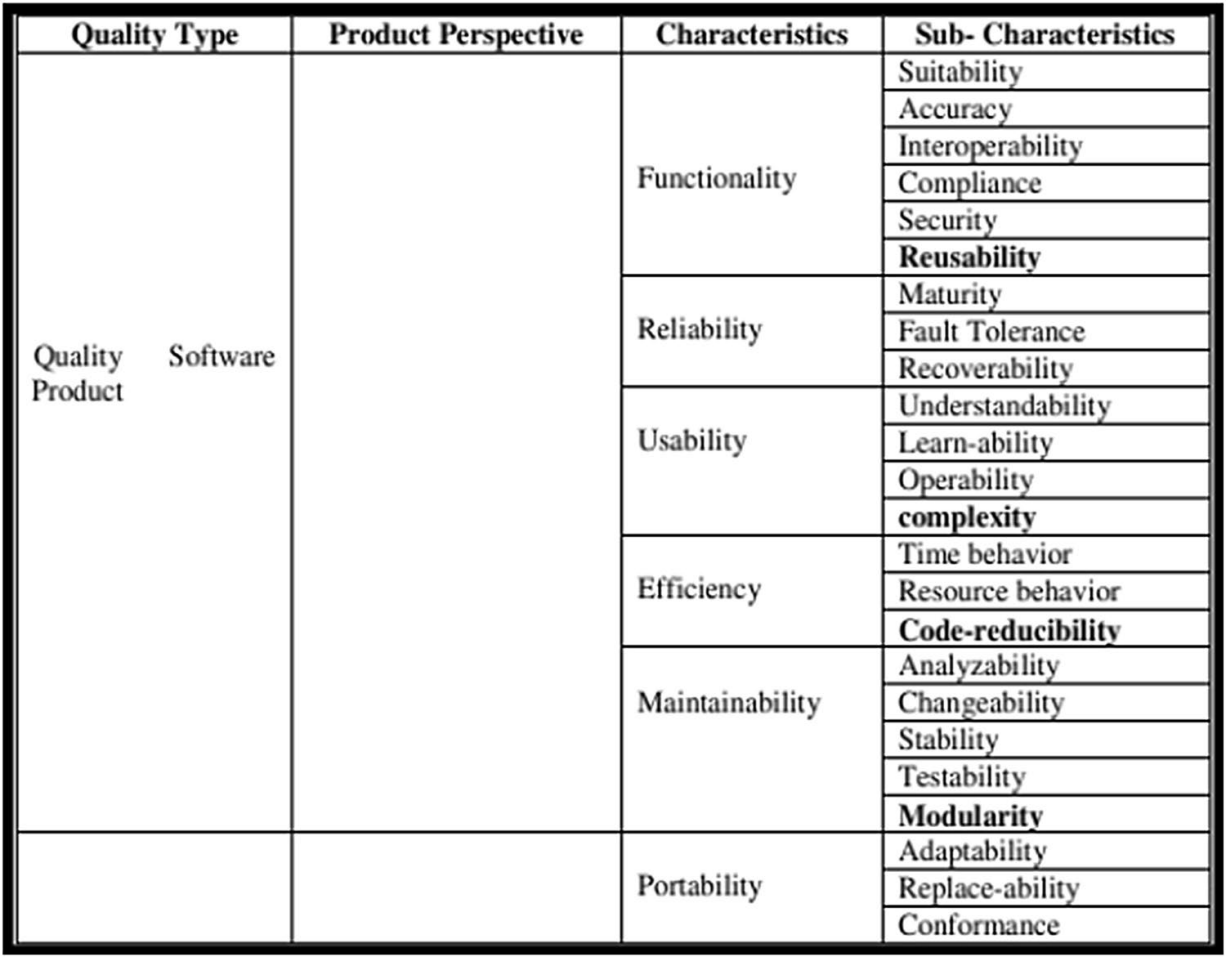
AOSQUAMO model and its contents^27^.
2. The Aspect-Oriented Software Quality (AOSQ) Model, which was proposed in 2012, was derived from the AOSQUAMO model and was intended for use in AOP-based applications. As shown in Fig. 6, the evolvability factor is added with four new sub-factors, which are sustainability, extensibility, design stability, and configurability^45^.

**Figure 6:**
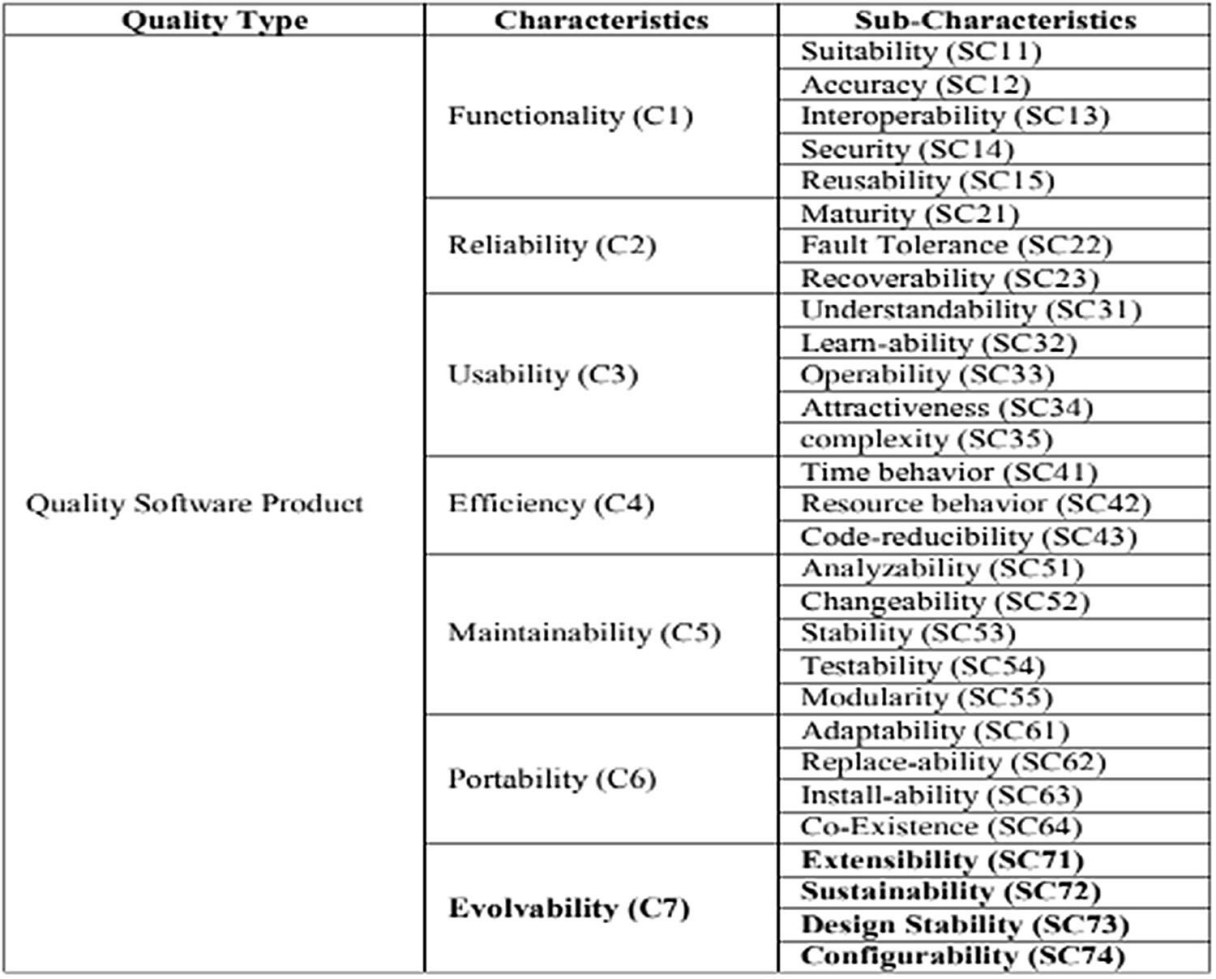
AOSQ model and its contents^45^.

### AOS Pitfalls and code smells

Some problems may be discovered in software, such as pitfalls, bugs, and code smells, and numerous researchers have studied them to reveal their effect on software quality in general and particularly on software reusability^3^. Studied the pitfalls in AOP and they concluded that some programmers’ mistakes occur at the documentation level of the software development process^46^. A catalog was made to document some pitfalls in OOP and AOP, based on the programmers’ expertise. The researchers tried to correlate these pitfalls, additionally to propose the Concern ReCS tool which alerts programmers to potential mistakes. Studied the topic of pitfalls as an effect on the AOS quality and reusability^44^, while^47^ included pitfalls as a negative effect on software product quality, and asserted that developers must take all possible steps to avoid them. According to our review of the existing literature, most studies have focused on OO software reusability. Less attention has been paid to AOS as there are few models dedicated to studying the effect of mistakes in AOS reusability. The mistakes that have been discovered and proposed are largely confined to OO software, although some of them are applicable on AOS. However, that isn’t enough because AOS fields of use are wide ranging, and there is a need to identify more metrics that are directly related to AOS reusability in order to make a good evaluation of AOS component reusability. This would make the software development process less time-consuming, since mistakes made during the early phases of SDLC, such the documentation or coding level can cause greater problems during the reuse process.

To overcome the above-mentioned problem, the current research set its focus on the possibility of estimating AOS reusability based on the pitfalls and bugs that can be discovered in the system and that negatively affect its quality and make it less reusable. The related attributes and metrics that have proven to be useful, as mentioned in the literature, were included, and some new attributes were proposed accordingly. The necessary metrics that affect AOS reusability have been included, and a new model has been built and proposed.

## Methodology and proposed approach

The main purpose of this study is to obtain an effective estimation of the reusability in AOS using a model that gives a valuable result depending on relevant attributes and metrics. The common metrics between OOP and AOP have been used in addition to others that are directly related to AOS reusability. To make the proposed model better and more effective than previously proposed models, new attributes like AOP pitfalls and generality that affect AOS reusability have been included alongside the important metrics and attributes which were proposed in the literature review. All metrics have been examined from various aspects and perspectives and consequently, a model that depends on the most significant ones has been proposed. Additionally, new metrics and attributes that affect the reusability of AO software have been taken into consideration.

AOS reusability has a strong effect on the software development process, as explained in the previous sections, and increases the possibility of a system’s components being suitable for reuse in the construction of new systems. Therefore, this study aims to answer the related research questions that seek an explanation for the most significant OOS reusability attributes and metrics that are applicable to AOS, and then use them to propose a new model that may especially affect AOS reusability. Finally, the study seeks to estimate AOS reusability at the package level using the proposed model which is based on the common applicable metrics and proposed AOS reusability metrics to test their effect on AOS reusability and determine which ones have a significant effect to obtain a new equation for AOS reusability estimation. The base for the equation is one of the popular equations that have been used in many previous studies to estimate AOS reusability. The SPSS program was used to analyze the results.

### Overall research design

**Figure 7:**
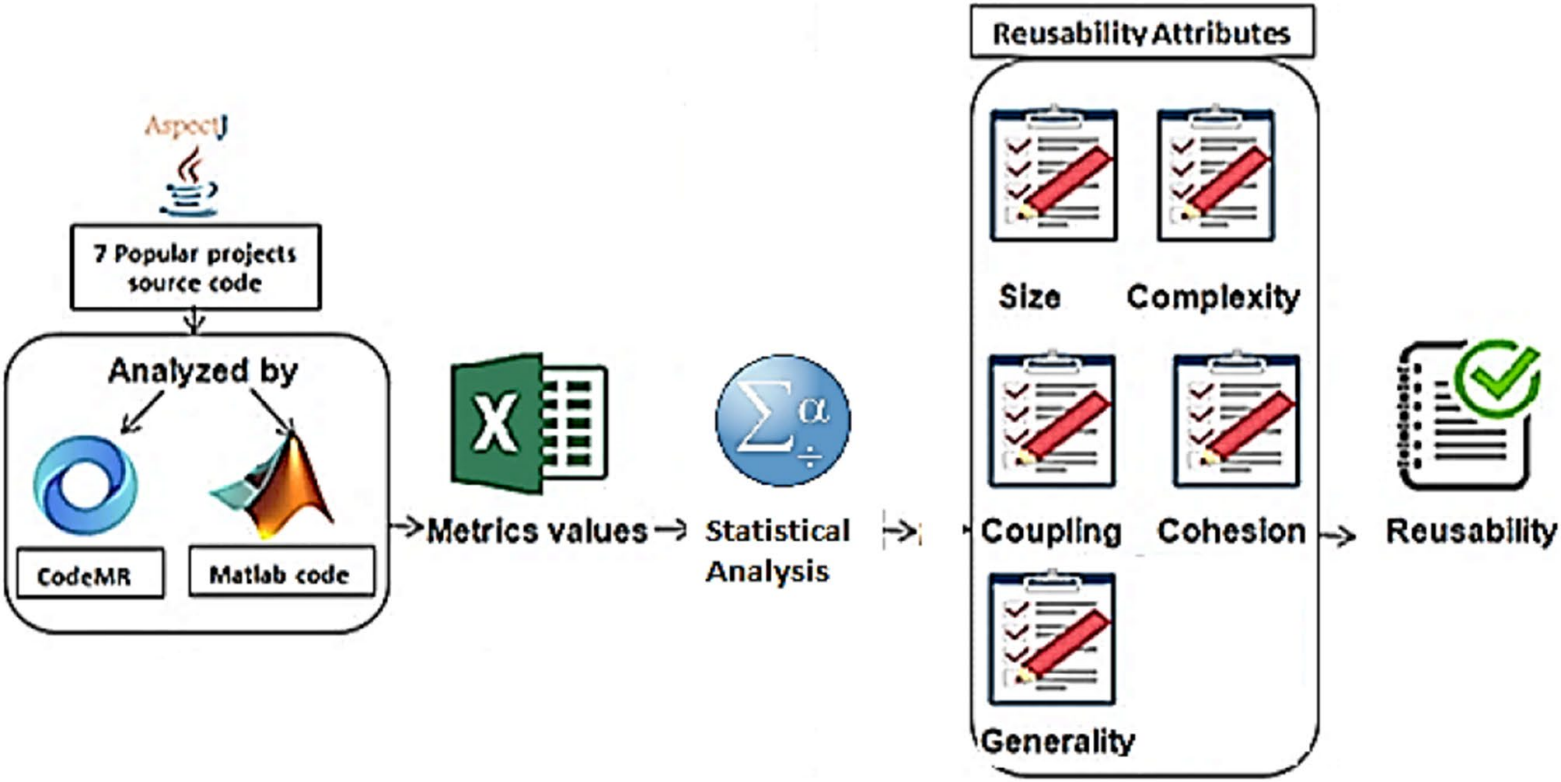
AOP reusability estimation model.

After studying and collecting AOS reusability metrics and attributes, and using many projects source code, and many programs to determine the most significant metrics that affect AOP reusability, AOS reusability model was designed to get new formula that used to calculate the reusability and get new values of it, as shown in Fig. 7.

Figure 7 shows the steps of AOP reusability estimation process that begins from selecting and choosing the projects which are seven projects that selected because they are the most popular and used in a lot of previous studies to evaluate their methods and models^10,45,48,49^, then the source codes of these projects analyzed by CodeMR eclipse plug-in (The results provided as a supplementary file with this research) and Matlab code (provided as a supplementary file with this research) to calculate the values of metrics affect AOP reusability, then Excel program used to save all metrics values for the source codes at many levels then make all values at package level by performing many calculations, and insert the old reusability formula to get the old reusability value for each package, then the reusability metrics values and old reusability values imported to SPSS program to make a statistical analysis of these values to obtain the metrics that strongly affect AOP reusability attributes, at the end a new formula obtained to get a new reusability value for each package.

### Research steps

- Step 1: We generally studied software reusability metrics and attributes according to related previous studies and their proposed models, and collecting them in a table to select which ones are maybe applicable to AOP or especially proposed for it.
- Step 2: We determined which ones are applicable to AOS and have been used to get their quality and reusability.
- Step 3: A search of metrics and attributes was conducted to identify those that are specifically designed for AOS reusability in order to calculate these metrics values based on many programs such as Eclipse and Matlab, and used these values to perform many processes.
- Step 4: The CodeMr plug-in was used to get the applicable metrics values of AspectJ projects source codes at different levels (The results provided as a supplementary file with this research) and saved them in Excel sheets to perform many calculations to make the values at package level.
- Step 5: An automated Matlab code (provided as a supplementary file with this research) was used to get the proposed metrics values of these packages which were saved in Excel sheets and used in many calculations.
- Step 6: The metrics values were obtained at package level and the old AOP reusability formula which obtained from previous studies and calculated based on these metrics values, then all results imported to SPSS program to be analyzed and to test their effect on AOP reusability.
- Step 7: Based on SPSS an analysis process performed on all metrics values and on the old AOS reusability formula results, which was proposed in previous studies, this research proposed a new AOP reusability estimation formula and a new values obtained of AOP reusability for each package after wrote it in Excel.

### Data set

The data set in this study is a set of AspectJ projects that were selected because they are open-source applications. They are easy to deal with, well known, and have been illustrated in many previous studies to test their models^10,45,48,49^. The projects were obtained from different websites like GitHub and SourceForge. These websites are available on the internet and have many AO systems designed by AspectJ. The list of 7 AO projects is HSQLDB, JHotDraw, Prevayler, Gtalkwap, Contract4j5, Ajefw, and Surrogate (all are provided as supplementary file with this research). The source code was also downloaded to perform an empirical experiment on the proposed model.

The projects that used are:

- **HSQLDB** (HyperSQL DataBase) is the leading SQL relational database system written in Java. It offers a small, fast multithreaded and transactional database engine with in-memory and disk-based tables and supports embedded and server modes.
- **JHotDraw** is a two-dimensional graphics framework for structured drawing editors that are written in Java. It is based on Erich Gamma's JHotDraw.
- **Prevayler** is an open source object persistence library for Java. It is an implementation of the Prevalent System design pattern.
- **Gtalkwap** this application is used for accessing Google chat service using WAP enable access. The application programmed by java, AspectJ languages.
- **Cntract4j5** is software gives sales, legal, financial, procurement, and contract administration teams an integrated set of tools for improved administration of their entire contract management approach.
- **Ajefw** (AspectJ Exception FrameWork) provides a central point and code reuse on the treatment of different kinds of exceptions presents in an application, everything specified in a xml configuration file.
- **Surrogate** is a predictive function that returns an estimated response value for a given set of numerical input parameters, which means with a given set of training data we can predict a response of our simulation for an unknown set of input parameters.

Integrating Aspect-Oriented Programming (AOP) into real-time applications poses several challenges. Performance overhead due to dynamic method interception can be prohibitive in systems where every millisecond counts. Complexity increases as AOP separates concerns, potentially obscuring the system's logic and making maintenance difficult. Timing issues arise as AOP may disrupt the strict timing requirements of real-time systems, potentially leading to missed deadlines. Resource constraints can also be problematic, with AOP frameworks consuming additional resources that may surpass system limits. Concurrency and synchronization concerns emerge, particularly in multithreaded environments, requiring careful management to maintain thread safety. Testing and verification become more challenging with AOP's introduction of new code paths and interactions. Dynamic aspect changes, often necessary in real-time systems, may not be easily accommodated by AOP frameworks, limiting adaptability. Mitigating these challenges necessitates a thorough understanding of the application's requirements and constraints, as well as thoughtful optimization and architectural decisions.

### Research tools

Several tools were adopted in this study. The most utilized essential tools and techniques are Matlab, SPSS, Eclipse, and Microsoft Excel. They served to analyze the metrics and attributes, implement the correlations between them, and provide a good estimation of reusability.

The programs that used are:

**Matlab** program used to write and run a Matlab codes (provided as a supplementary file with this research) that calculate the proposed metrics values.

**SPSS** is a statistical analysis program that used to perform some statistics processes on all metrics values and on the old AOP reusability values to obtain the most significant metrics that affect AOP reusability and formulate a new formula to estimate AOP reusability.

**Eclipse** program used to calculate the applicable metrics values based on CodeMr plug-in (The results provided as a supplementary file with this research) which specifically support AspectJ language.

**Excel** program used to save all metrics values and calculate them at package level, then calculate the old and new AOP reusability values after insert their formulas.

## AOS reusability metrics and attributes

According to the related literature, six attributes and 21 metrics have been included because they have a significant effect on the AOS reusability. 17 of the metrics are OOS reusability metrics and applicable on AOS. They can be found in the studies by^24^^47,50^, and comprise: **#IC** (Number of Interfaces and classes), **NOM** (Number of Methods), **NOF** (number of fields (attributes) in a class), **WMC** (Weighted Method Count), **CBO** (Coupling Between Object Classes), **LCOM** (Lack of Cohesion of Methods)**, NOC** (Number of Children), **NORM** (Number of Overridden Methods), **SI** (Specialization Index), **RFC (**Response for a Class), **DIT (**Depth of Inheritance Tree), **EC** (Efferent Coupling), **ATFD** (Access to Foreign Data), **AC** (Afferent Coupling), **LTCC** (Lack of Tight Class Cohesion), **LCAM** (Lack of Cohesion Among Methods), **ABS** (Abstractness). 4 of the metrics are especially tailored for AOS^10,44,46,47^, and they are: **#Aspects, #Pointcuts, #Public Classes, #Pitfalls**. These metrics were measured by CodeMR (The results provided as a supplementary filewith this research) and the automated Matlab code (provided as a supplementary file with this research). The values were then inserted to Excel sheets to apply the reusability equations and some calculations on them. The obtained results were imported to the SPSS program to be analyzed, and the most significant attributes and metrics which affect AOS reusability were obtained (see Table 2). These attributes and metrics result from the SPSS analysis process and each one has a calculated effect constant that will be explained in the “Results and evaluation” section.

**Table 2:** AOS reusability attributes and metrics.

The attribute	The metric	Definition
Size	#IC	Total number of Interfaces and classes
	NOM	Number of Methods in a class
	NOF	Number of Fields (attributes) in a class
	#Aspects	Number of Aspects
	WMC	Weighted Method Count
	#pointcuts	Number of points cuts
Complexity	CBO	Coupling Between Object Classes The number of classes that a class is coupled to is calculated by counting other classes whose attributes or methods are used by a class, plus those that use the attributes or methods of the given class
	LCOM	Lack of Cohesion of Methods Measure how methods of a class are related to each other
	NOC	NOC Number of Children The number of direct subclasses of a class
	NORM	Number of Overridden Methods
	WMC	Weighted Method Count
	SI	Specialization Index
	RFC	Response For a Class
	DIT	Depth of Inheritance Tree
Coupling	EC	EC Efferent Coupling Outgoing Coupling. The number of classes in other packages that the classes in the package depend upon is an indicator of the package’s dependence on externalities
	ATFD	Access to Foreign Data is the number of classes whose attributes are directly or indirectly reachable from the investigated class
	AC	Afferent Coupling Incoming Coupling. The number of classes in other packages that depend upon classes within the package
	CBO	Already Defined
	NOC	Already Defined
Cohesion	LTCC	The Lack of Tight Class Cohesion metric measures the lack cohesion between the public methods of a class. That is the relative number of directly connected public methods in the class
	LCAM	Lack of Cohesion Among Methods (1-CAM) CAM metric is the measure of cohesion based on parameter types of methods. LCAM = 1-CAM
	LCOM	Already Defined
Generality	#Public classes	Already Defined
	AC	Already Defined

The generality attribute (shown in Table 2) is a new AOS reusability attribute that is illustrated in this study with its metrics. This attribute has been proposed in previous studies for the software in general and for OOS^6,15,51,52^. It was mentioned by Chaudhary and Chatterjee in^53^ but it has yet to be evaluated. The most significant metrics used to evaluate it in this study are **AC** (Afferent Coupling) and **#Public classes**.

There are many applicable metrics that have been proposed in this study and considered in Eq. (2), that weren’t considered in Eq. (1) as to their effects on its original attributes. These metrics were tested, studied, and analyzed by SPSS program, as to whether they affect reusability or not, and based on the testing results, we found that they affect the AOS reusability. These applicable metrics are **CBO**, **AC**, **NOC**, and **LCOM**.

## AOS reusability estimation

Many previous studies^18,36,44,54^ used Eq. (1) to estimate AOS reusability based on specific metrics and attributes, but each study focused on one metric or attribute, or a very few of them, so failed to give a comprehensive value of AOS reusability, due to the use of an incomplete model. This equation was used to get a supported value that is illustrated in the evaluation of the results of this study. The old values of AOS reusability have been used in the comparison process of the new AOS reusability values.

1 $$ {\mathbf{old}} {\mathbf{Reusability}} = - 0.25 {\mathbf{x}} {\mathbf{Coupling}} + 0.25 {\mathbf{x}} {\mathbf{Cohesion}} + 0.5 {\mathbf{x}} {\mathbf{Messaging}} + 0.5 {\mathbf{x}} {\mathbf{Design}} {\mathbf{Size}} $$

Equation (1) is based on the attributes and metrics that have been explained in the previous section for old reusability calculation. After obtaining the old reusability values based on Excel program, SPSS program was used to analyze the results to study the correlation between them and the metric values to get the effect constant of each metric that affects AOS reusability. Based on these constants, a new reusability equation for ASO is built with new metrics.

2 $$ \begin{aligned} {\mathbf{New}} {\mathbf{Reusability}} & = - 1.33* {\mathbf{NOM}} - 45.18* \# {\mathbf{Aspects}} + 2.26* {\mathbf{NORM}} - 25.98*{\mathbf{SI}} \\ & \;\;\; - 91.29*{\mathbf{ATFD}} + 1.38* {\mathbf{CBO}} - 12.49* {\mathbf{NOC}} - 39.32*{\mathbf{LCAM}} \\ & \;\;\; + 46.81* {\mathbf{LCOM}} + 0.46*{\mathbf{RFC}} + 26.64* \# {\mathbf{IC}} \\ \end{aligned} $$

Each metric has a correlation with one or more of the five attributes that are explained in Table 2, and has a specific effect constant on the reusability that was obtained based on the analysis and calculations results to be explained and discussed in section “AOS reusability estimation”.

The new reusability equation has new metrics. Some of them were used in the old reusability equation and some of them have been proposed in this study. Thus, Eq. (2) is more comprehensive than Eq. (1), because it considered most of the metrics that affect AOS reusability, and gives us valuable results for AOS reusability.

## Results and evaluation

Out of the 7 projects taken in the research as a case study application, in this section, we will present only the **Prevayler project** analysis.

### The results of Prevayler project analysis

**Figure 8:**
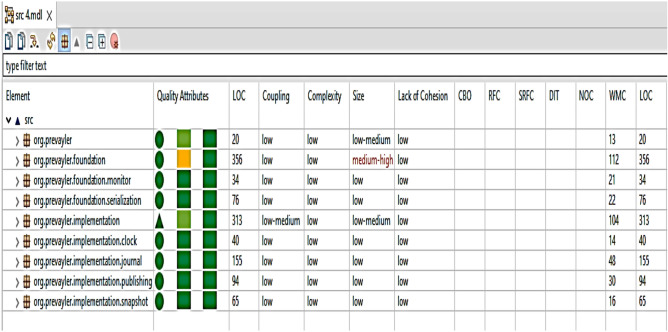
The applicable metrics at package in Prevayler project.

CodeMR results (provided as a supplementary file with this research) contain graphical and numerical information about the Prevayler project and its packages. The applicable metrics values were calculated at different levels, which are method level, class level, and package level. Figure 8 shows values of some metrics at package level such as (WMC, #(IC), AC, EC, and Abs).

**Figure 9:**
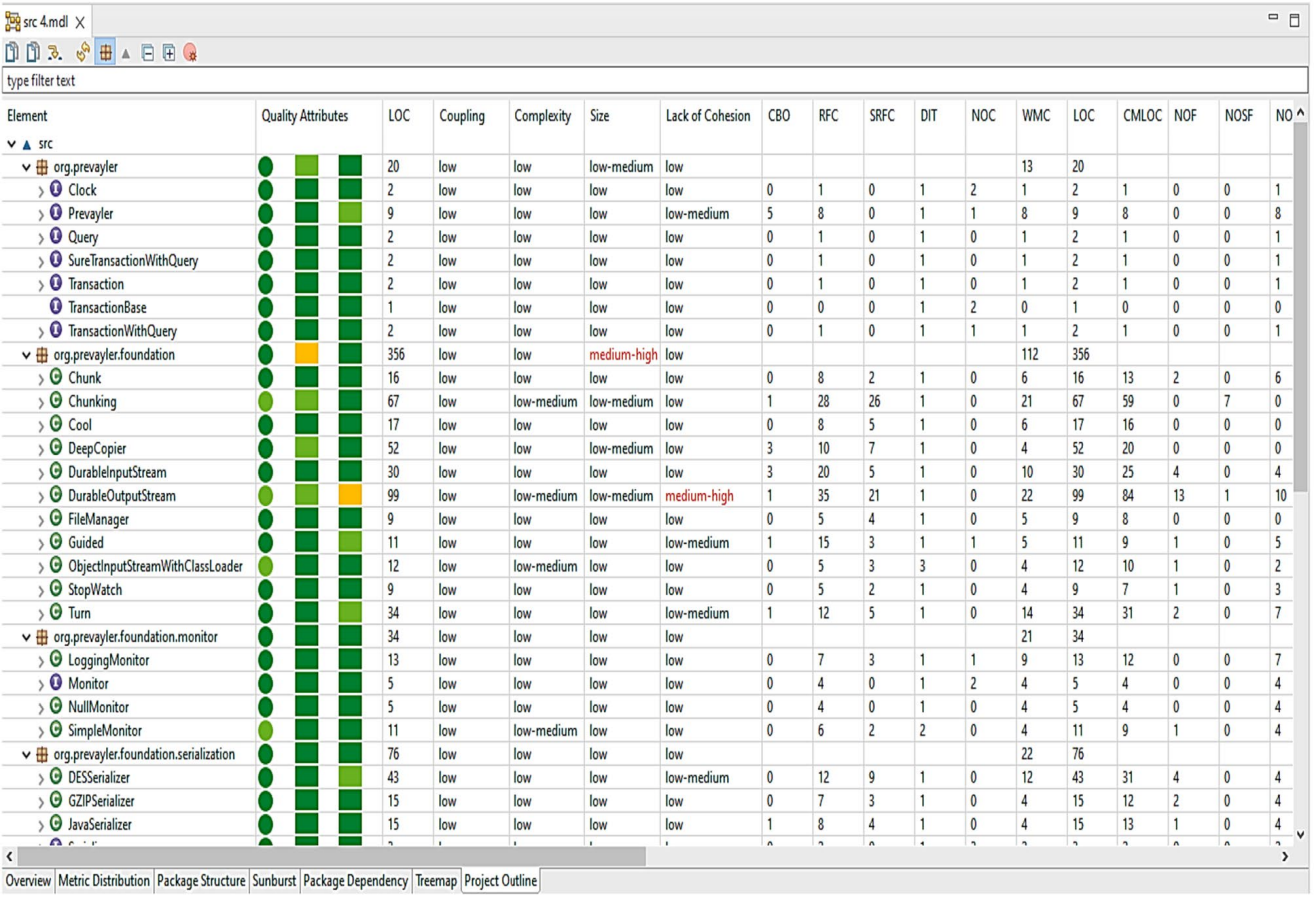
The applicable metrics at class and method level in Prevayler project.

Figure 9 shows values of some metrics at class and method level such as (CBO, RFC, DIT, NOC, WMC, NOF, NOM, LCOM, LCAM, LTCC, ATFD, and SI).

**Figure 10:**
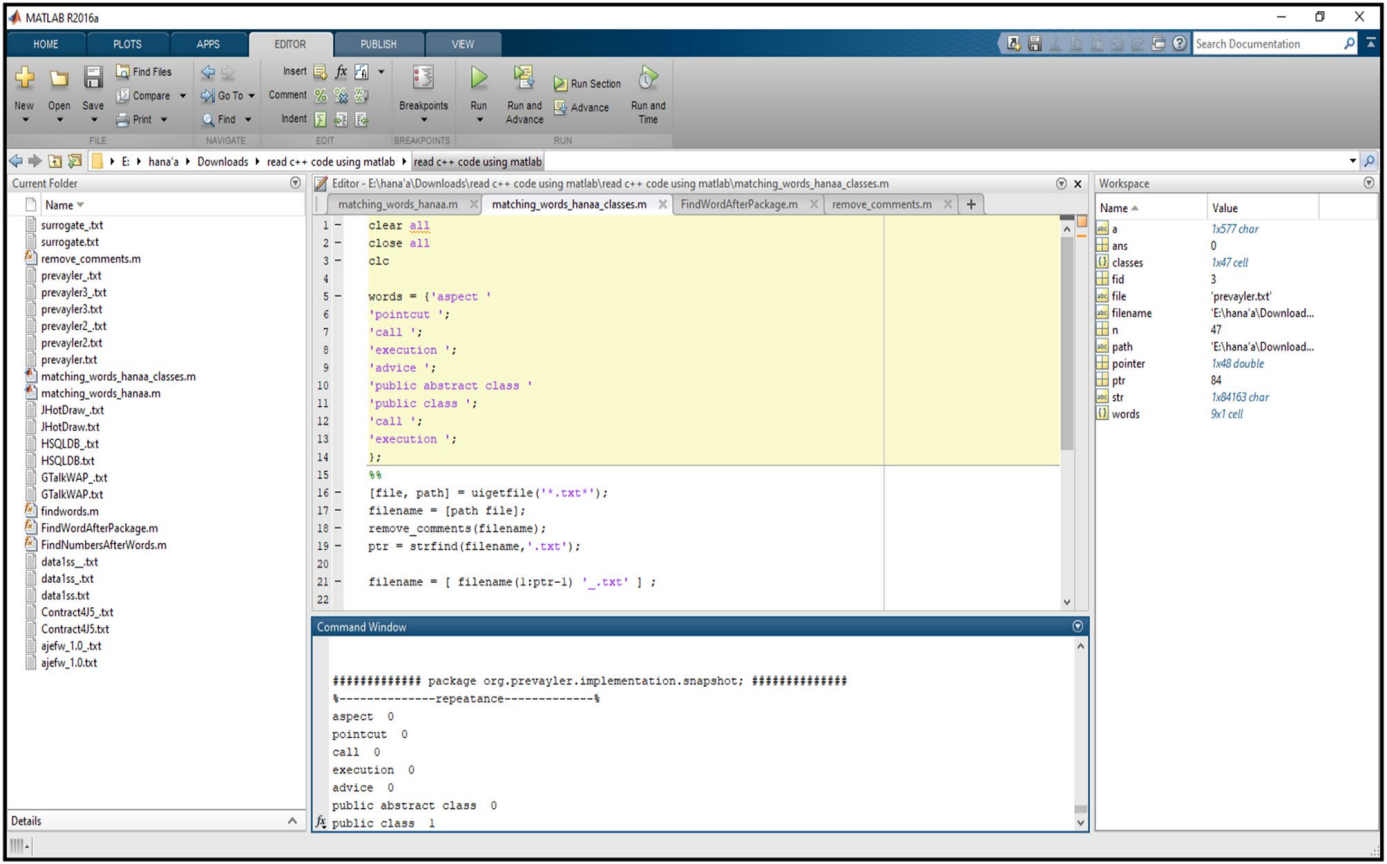
The proposed metrics at package level in Prevayler project.

An automated Matlab code (provided as a supplementary file with this research) was used to calculate the proposed metrics, which are **#Aspects, #Pointcuts, #Public Classes, #Pitfalls** for the Prevayler project (shown in Fig. 10).

The CodeMR and Matlab code (provided as a supplementary file with this research) results (The results provided as a supplementary file with this research) were stored in Microsoft Excel sheets and used in some calculations to find the old reusability values for each package in all projects, which are 7 projects with 62 packages. The SPSS program was used to analyze the metrics values and study the correlations between them and the old reusability values to get an effect constant for each metric under consideration.

After the linear regression was performed, the important results shown in Table 3 and Table 6 were obtained. Table 3 contains the entered and removed metrics, and Table 6 contains the effect constant for each metric.

**Table 3:** The entered and removed metrics.

Variables entered/removed^a^			
Model	Variables entered	Variables removed	Method
1	#IC, # pitfalls, # Aspects, AC, ABS, ATFD, #public classes, CBO, SI, # pointcuts, NORM, NOF, NOC, WMC, DIT, NOM, EC, LTCC, RFC, LCOM, LCAM, WMC^b^		Enter

^a^Dependent variable: reusability.

^b^Tolerance = 0.000 limit reached.

As is clear in Table 3, all entered metrics were entered and none were removed, which means that all metrics are important. The dependent variable is the old reusability value which has been illustrated as a base to perform the analysis process.

Regression analysis was performed using the SPSS tool, where the values of reusability used as a dependent variable and the other metrics (Object-oriented and Aspect-oriented metrics) values were used as independent variables.

The following three tables highlight the results of the regression analysis process. Table 4 shows the values of R (multiple correlation coefficient) and $R^{2}$, and the standard error of the estimate that were used to determine how well a regression would fit the data. The value of R in the applications used in this experiment is 0.999. This value indicates a very good level of prediction. The value of $R^{2}$ is 0.998 which means that 99.8% of the variability observed in the target variable is explained by the regression model.

**Table 4:** Model summary from SPSS as a result of running the applications.

Model	R	R square	Adjusted R square	Std. error of the estimate
1	0.999^a^	0.998	0.997	51.376641255344886

^a^Predictors: (Constant), #IC, # pitfalls, # Aspects, AC, ABS, ATFD, #public classes, CBO, SI, # pointcuts, NORM, NOF, NOC, WMC, DIT, NOM, EC, LTCC, RFC, LCOM, LCAM, WMC.

Table 5 indicates whether the regression analysis process is a good fit for the data or not. It shows that the independent variables are a statistically significantly prediction of dependent variable, where the significant value is close to 0.0 (1.490E−046) which is less than 0.05. We should mention here that the threshold value for significance is 0.05. If the value of significance is less than 0.05 (p) then we include the value of the referred metrics. Table 6 shows the coefficient values for the measured metrics, for the applications.

**Table 5:** ANOVA Table from SPSS as a result of running the 3 applications.

ANOVA^a^						
Model		Sum of squares	df	Mean square	F	Sig
1	Regression	55,016,301.399	22	2,500,740.973	947.409	1.490E-046^b^
	Residual	102,942.811	39	2639.559		
	Total	55,119,244.211	61			

^a^Dependent variable: reusability.

^b^Predictors: (Constant), #IC, # pitfalls, # Aspects, AC, ABS, ATFD, #public classes, CBO, SI, # pointcuts, NORM, NOF, NOC, WMC, DIT, NOM, EC, LTCC, RFC, LCOM, LCAM, WMC.

**Table 6:** Coefficients table from SPSS as result of running the three applications.

Coefficients^a^						
Model		Unstandardized coefficients		Standardized coefficients	t	Sig.
		B	Std. error	Beta		
1	(Constant)	− 24.131	13.313		− 1.813	0.078
	NOM	− 1.326	0.299	− 0.272	− 4.436	0.000
	NOF	0.260	1.017	0.009	0.256	0.799
	WMC	0.436	0.334	0.554	1.306	0.199
	# pointcuts	29.368	20.443	0.020	1.437	0.159
	# Aspects	− 45.183	19.221	− 0.031	− 2.351	0.024
	WMC	0.111	0.709	0.070	0.157	0.876
	NORM	2.261	0.865	0.073	2.615	0.013
	SI	− 25.984	4.144	− 0.162	− 6.270	0.000
	DIT	− 1.819	1.262	− 0.046	− 1.441	0.157
	EC	− 2.931	5.332	− 0.026	− 0.550	0.586
	ATFD	− 91.291	9.717	− 0.227	− 9.395	0.000
	CBO	1.382	0.055	0.512	25.296	0.000
	NOC	− 12.486	4.641	− 0.077	− 2.690	0.010
	LTCC	3.453	12.406	0.017	0.278	0.782
	LCAM	− 39.321	18.904	− 0.252	− 2.080	0.044
	LCOM	46.805	13.030	0.217	3.592	0.001
	#public classes	1.060	1.027	0.010	1.032	0.308
	RFC	0.458	0.141	0.244	3.254	0.002
	ABS	25.647	35.900	0.007	0.714	0.479
	AC	− 4.458	4.167	− 0.011	− 1.070	0.291
	# pitfalls	3.098	5.298	0.005	0.585	0.562
	#IC	26.643	6.301	0.295	4.229	0.000

^a^Dependent variable: reusability.

Table 6 shows that the most influential metrics (based on significant values) are **NOM, #Aspects, NORM, SI, ATFD, CBO, NOC, LCAM, LCOM, RFC, IC** for which the significant values are less than 0.05. For example, the effect of **NOM** metric (b = − 1.326, p = 0.000) is significant and its coefficient is negative, indicating that the greater the number of methods in a system, the more difficult it is to reuse.

Table 6 shows the effect constant for each metric which appears next to it, and the new reusability equation that has been built in the previous section based on the metrics that their (t) value isn’t zero, which means it positively or negatively affects the reusability. (**NORM, CBO, LCOM, RFC, IC**) positively affect reusability by (2.26, 1.38, 46.81, 0.46, and 26.64) respectively. **(NOM, #Aspects, SI, ATFD, NOC, LCAM**) negatively affect reusability by (1.33, 45.18, 25.98, 91.29, 12.49, and 39.32) respectively.

The new reusability equation of AOS was inserted to the Excel sheet, and a new reusability value was calculated for each package as shown in Table 7. There is a difference between the reusability values that indicates the impact of the generality and complexity attributes which have been included in the new equation.

**Table 7:** The old and new AOS reusability values.

Package name	Old reusability	New reusability
ua.cn.gtalkwap	2	28.02
ua.cn.gtalkwap.aspects	6	25.77
ua.cn.gtalkwap.core	17	52.41
ua.cn.gtalkwap.web.bean	57.25	54.4
ua.cn.gtalkwap.web.renderer	22.5	25.77
ua.cn.gtalkwap.web.taglib	11	26.64
ua.cn.gtalkwap.web.util	39.5	53.28
ua.cn.gtalkwap.web.wml	13.75	39.92
gj.ajframework	85.19775	85.81716
gj.app	76.35775	100.56361
gj.errormod	81.32625	182.24959
java.net.sf.surrogate.core	90.4165	51.85288
resources.jasmin.net.sf.surrogate.core	1.5	26.64
CH.ifa.draw.applet	150.2375	149.63051
CH.ifa.draw.application	215.7115	215.19588
CH.ifa.draw.contrib	425.5135	306.81254
CH.ifa.draw.figures	881.878	796.33309
CH.ifa.draw.framework	182.8545	99.50424
CH.ifa.draw.samples.javadraw	205.17675	252.32876
CH.ifa.draw.samples.net	46.19725	82.66876
CH.ifa.draw.samples.nothing	12.111	104.05192
CH.ifa.draw.samples.pert	131.35275	131.9427
CH.ifa.draw.standard	994.8495	918.14111
CH.ifa.draw.util	473.3385	462.51283
org.hsqldb.auth	327.708	180.08779
org.hsqldb.cmdline	2472.8085	1012.50477
org.hsqldb.cmdline.sqltool	594.32	226.45035
org.hsqldb.dbinfo	3045.39925	2137.67523
org.hsqldb.error	282.846	121.25284
org.hsqldb.index	947.88275	408.43602
org.hsqldb.jdbc	4657.573	2047.28331
org.hsqldb.jdbc.pool	233.76725	258.91968
org.hsqldb.lib.java	17.6375	56.884
org.hsqldb.lib.tar	740.119	368.86688
org.hsqldb.map	914.7735	283.56692
org.hsqldb.navigator	597.9875	208.80641
org.hsqldb.resources	36.93025	49.89064
org.hsqldb.result	902.12125	516.83487
org.hsqldb.rights	835.85425	265.17559
org.hsqldb.rowio	962.561	427.38826
org.hsqldb.sample	176.68425	109.70357
org.hsqldb.scriptio	366.18	243.76763
org.hsqldb.server	1960.3985	1151.21322
org.hsqldb.test	416.90675	222.37027
org.hsqldb.types	4530.15075	1230.30978
org.hsqldb.util.preprocessor	613.8025	305.07185
org.prevayler	13.925	-94.214
org.prevayler.foundation	216.87025	208.76592
org.prevayler.foundation.monitor	29.7965	6.84648
org.prevayler.foundation.serialization	49.7125	11.95671
org.prevayler.implementation	176.88475	232.84297
org.prevayler.implementation.clock	30.639	71.52658
org.prevayler.implementation.journal	85.97925	106.21416
org.prevayler.implementation.publishing	52.523	166.53974
org.prevayler.implementation.snapshot	37.74475	34.15818
org.contract4j5.configurator	12	78.1
org.contract4j5.controller	2	28.02
org.contract4j5.enforcer	100.47675	66.38881
org.contract4j5.enforcer.defaultimpl	5.83325	14.56644
org.contract4j5.errors	6	25.77
org.contract4j5.testexpression	12	52.41
org.contract4j5.utils	5	26.64

### Discussion and evaluation

To mitigate the risk of overfitting and to ensure the reliability of correlation coefficients, we used cross-validation techniques to assess the stability and generalizability of correlation estimates across different subsets of the data. We evaluated the consistency of correlation coefficients using a 20% sample and an 80% sample. As we can see from Table 8 below, the correlation coefficient calculated using 20% sample (0.969) is slightly higher than the correlation coefficient calculated using an 80% sample (0.921). Therefore, the correlation coefficients between the two subsets of the data (20% sample and 80% sample) do not vary widely, indicating no overfitting.

**Table 8:** Correlations table from SPSS as result of running the three applications.

Correlations				
Sample			Reusability	Predicted
20% Sample	Reusability	Pearson correlation	1	0.969**
		Sig. (2-tailed)		0.001
		N	6	6
	Predicted	Pearson correlation	0.969**	1
		Sig. (2-tailed)	0.001	
		N	6	6
80% Sample	Reusability	Pearson correlation	1	0.921**
		Sig. (2-tailed)		0.000
		N	56	56
	Predicted	Pearson correlation	0.921**	1
		Sig. (2-tailed)	0.000	
		N	56	56

**Correlation is significant at the 0.01 level (2-tailed).

Based on the results, we can draw the following conclusions:

- With 20% sample the old reusability and new reusability have a statistically significant linear relationship (r = 0.969, p < 0.001).
- With 80% sample the old reusability and new reusability have a statistically significant linear relationship (r = 0.921, p < 0.001).
- The direction of the relationship is positive, meaning that old reusability and new reusability are positively correlated. This indicates that as old reusability increases, new reusability tends to increase as well.
- The magnitude, or strength, of the association is relatively high (| r |> 0.9).

The reusability values which have been calculated and appeared for each package in the previous section in Table 9 are divided into two columns. The first one has the old reusability values of AOS, calculated based on Eq. (1), which was constructed in the previous studies. The second column has the new reusability values of AOS, calculated based on Eq. (2), which has been proposed in this study.

**Table 9:** The coefficient correlation between reusability and each metric.

The Metric	The metric’s impact on new reusability
#(IC)	0.633568573
NOM	0.845762729
NOF	0.801471253
# pointcuts	− 0.084698476
# Aspects	− 0.084244047
CBO	0.899260295
LCOM	0.764050092
WMC	0.96575989
NOC	0.509101881
NORM	0.602049373
SI	0.355607905
RFC	0.914884093
DIT	0.554874776
EC	0.555604317
ATFD	0.725972978
AC	0.076133547
LTCC	0.734987037
LCAM	0.699108086
#Public classes in the package	0.427785418
Abs	− 0.133958274
# Pitfalls	0.037880461

Many original metrics included in Eq. (1) have also been included in Eq. (2). The affect constant of each one that impacts AOS reusability was based on SPSS analysis results, which are as follows:

1. **Coupling** was affected by (**ATFD** (- 91.29), **CBO** (1.38), **NOC** (- 12.49)).
2. **Cohesion** was affected by (**LCAM** (-39.32), **LCOM** (46.81)).
3. **Complexity** was affected by (SI (-25.89), **CBO** (1.38), LCOM (46.81), NOC (12.49), RFC (0.46), NORM (2.26)).
4. **Design size** was affected by (#IC (26.64), #Aspects (-45.18)).

The attributes that were added to Eq. (2) are **complexity** which has been affected by (**SI** (− 25.89), **CBO** (1.38), **LCOM** (46.81), **NOC** (12.49), **RFC** (0.46), **NORM** (2.26)), and **generality** which has an effect on AOS reusability based on two influential metrics (**#Public classes** (1.06), and **AC** (− 4.458)). These two metrics indicate the popularity degree of the package, because if the number of public classes in the package is high, it means that the package’s functions are available and ready to be called and illustrated by the external objects. In turn, this means that the functions in the package have a high expected popularity by the developers who built the system. This result indicates that this package has a high potential for reuse in new systems that have similar functionality, and that makes the reusability of it high.

Table 8 shows the correlation coefficient values between reusability and all proposed metrics. Based on it, there are some metrics that negatively affect the AOS reusability, such as **#Pointcuts**, **#Aspects**, and **Abs,** that lead to an increase in the complexity and size of the software. On the other hand, they have a positive impact which may lead to an increase in cohesion and a decrease in coupling. Other metrics have a positive effect on AOS reusability, such as **AC** (Afferent Coupling) and **#Public classes** because they may lead to an increase in the generality of the component, which makes it highly suitable for reuse. **CBO**, **NOC**, and **DIT** are metrics that have a negative effect on reusability because if they are high it leads to an increase in the coupling, complexity, and size. So these metrics have an important role in the reusability values difference.

**Figure 11:**
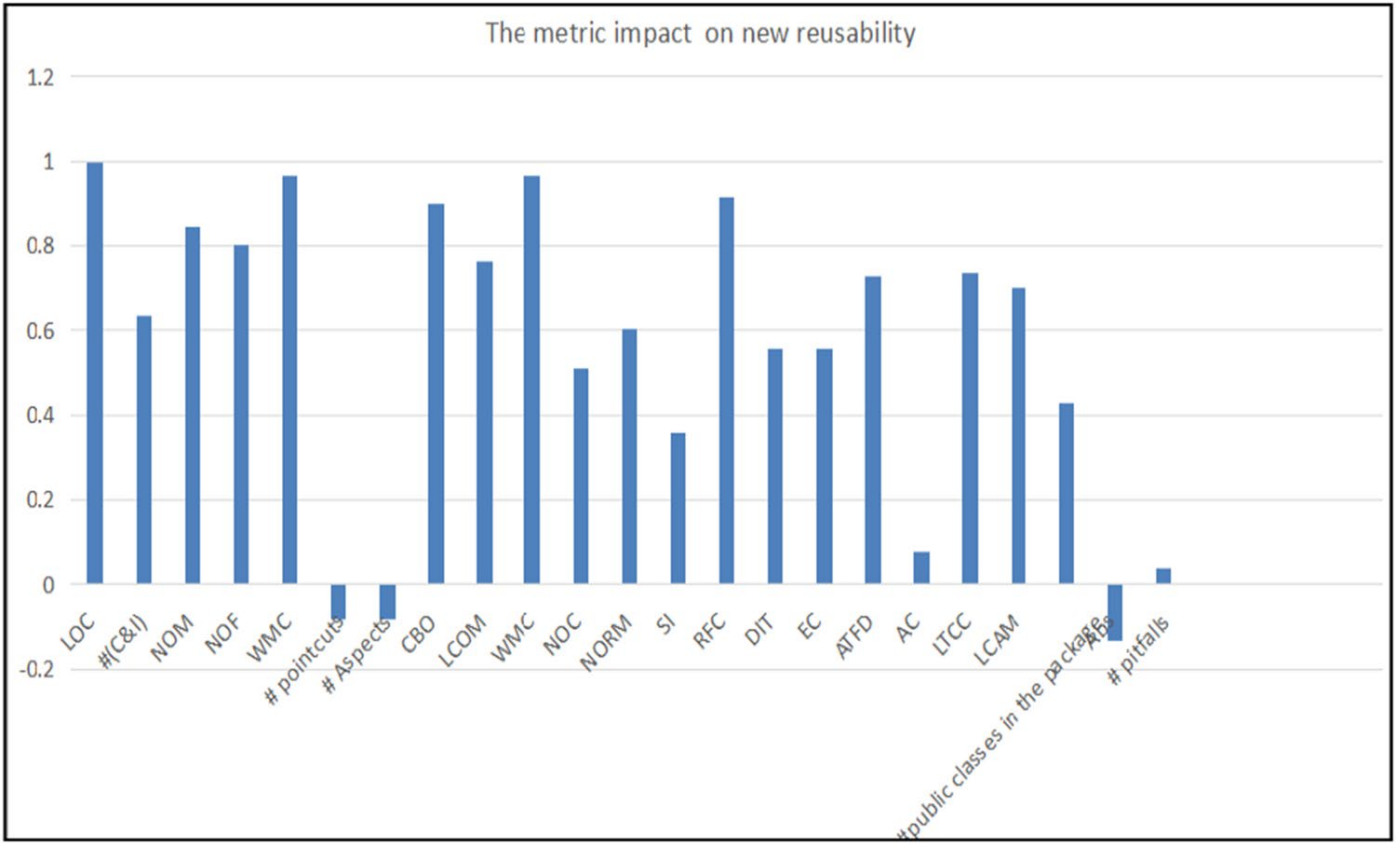
The metrics’ impact of new reusability.

Figure 11 shows the affect strength of each metric on reusability, and as we have noted, the most significant metrics that positively affect reusability are (LOC, WMC, CBO, and RFC), and the most significant metric that negatively affects reusability is (#Public classes). There are some metrics that have a weak impact on reusability, such as (#Pitfalls, AC, #Pointcuts, and #Aspects). Some metrics were removed during the analysis process carried out using the SPSS program.

## Conclusion and future work

In this study, we worked on collecting the AOS reusability attributes and metrics and then calculated their values based on CodeMR tool (The results provided as a supplementary file with this research), an automated Matlab code (provided as a supplementary file with this research), and Microsoft Excel. An analyzing process was performed on their values using the SPSS program to get the correlation between the metrics values and the old reusability values. The new equation of AOS reusability that was obtained depended on the effect constant value for each metric. The calculated values of old reusability and new reusability were different in some packages, because the values of the proposed metrics are different among the packages. In some packages, the values increased because the metrics values have a positive effect more than the metrics values which have a negative effect. In other packages, the new reusability value is decreased because the metrics values which have a negative affect are more than the metrics values which have a positive effect, and in some packages, the new reusability value hasn’t been changed, because the proposed metrics values are zeros. Some new metrics which were proposed have a positive effect on the AOS reusability attributes, such as **#Public classes** and **AC** (Afferent Coupling), and some of them have a negative effect on its attributes, such as **EC** (Efferent Coupling), **NOC** (Number of Children) and others that have been explained.

In this work, the generality attribute has been studied and proposed as an effect on AOS reusability, in addition to many metrics that affect an existing AOS reusability attribute such as #Public classes, AC (Afferent Coupling), EC (Efferent Coupling), and NOC (Number of Children) but there are potential influencers that may effect on AOS reusability, such as software domain, code pitfalls, and smells. In the future, researchers must be concerned about the software domain, and study the correlation between it and reusability. There is also a need for further studies on the code pitfalls and smells effect, because they may have a strong effect on the reusability of the software, as has been mentioned and discussed in some previous studies.

The proposed model for AOS reusability estimation is comprehensive compared to previous models, because the previous ones were concerned with specific metrics. The results obtained from this study have revealed the most significant metrics and attributes that affect AOS reusability. Thus, the new values of reusability are more valuable compared to the old values, which helps software developers to take the correct decision about the component that will be reused in the new system at a lower development cost.

The findings of this study hold significant implications for advancing the assessment and measurement of Aspect-Oriented Systems (AOS) reusability. The development of a novel model for AOS reusability estimation, based on a new equation incorporating five attributes, marks a substantial contribution to the field. Notably, this equation considers three attributes—coupling, cohesion, and design size—previously studied in the literature, while introducing complexity and generality as new dimensions to be evaluated. Each attribute is measured using metrics proposed in this study, enriching the evaluation framework. By applying the proposed equation to seven aspect projects as case studies, this research demonstrates the practical applicability of the model. The emergence of new reusability values compared to those derived from the previous equation underscores the significant impact of the proposed reusability metrics and attributes. This indicates the efficacy of the model in capturing a more nuanced understanding of AOS reusability.

Moreover, the inclusion of complexity and generality as attributes enriches the reusability assessment process, providing developers with a more comprehensive understanding of factors influencing reuse potential. This broader scope enables informed decision-making regarding component reuse, potentially leading to more efficient and cost-effective software development practices. Overall, the development and application of the proposed model represent a significant advancement in the field of AOS reusability assessment. By incorporating new attributes and metrics, the model enhances the accuracy and relevance of reusability evaluations, thereby facilitating improved decision-making and contributing to the advancement of software development practices.

In the future, our research would be directed towards (1) Identifying Reusable Aspects by analyzing AO codebases using reusability metrics, academics and research can find aspects that are reusable across various applications, (2) Evaluating Aspect Design Patterns by AO metrics it can be used to estimate the effectiveness of different aspect design patterns in endorsing reusability and finally (3) Benchmarking Reusability since AO metrics can serve as benchmarks for comparing the reusability of AO code with code written using other programming paradigms.

### Supplementary Information

Supplementary Information.

## Data Availability

Data is available on request due to privacy/ethical restrictions. The data that support the findings of this study are available on request from the corresponding author, Dr. Aws Magableh, or can be taken from the details given in the code availability section of the method.

## Code availability

The readers can access the code, algorithm, project and data sets without any restrictions. To ensure that other researchers can access it and make use of it to replicate the work if needed. All Supplementary document of this research work has been published to Zenodo repository.

## References

[1] Frakes WB, Kang K (2005). Software reuse research: Status and future. IEEE Trans. Softw. Eng..

[2] Sahu K, Srivastava RK, Kumar S, Saxena M, Gupta BK, Verma RP (2023). Integrated hesitant fuzzy-based decision-making framework for evaluating sustainable and renewable energy. Int. J. Data Sci. Anal..

[3] Sahu K, Srivastava RK (2021). Predicting software bugs of newly and large datasets through a unified neuro-fuzzy approach: Reliability perspective. Adv. Math. Sci. J..

[4] Mäkitalo N, Taivalsaari A, Kiviluoto A, Mikkonen T, Capilla R (2020). On opportunistic software reuse. Computing.

[5] Zaragoza MG, Kim HK (2018). Mobile application development on domain analysis and reuse-oriented software (ROS). Stud. Comput. Intell..

[6] Aggarwal J, Kumar M (2021). Software metrics for reusability of component based software system: A review. Int. Arab J. Inf. Technol..

[7] Haefliger S, Von Krogh G, Spaeth S (2008). Code reuse in open source software. Manage Sci..

[8] Maras J, Štula M, Crnkovíc I (2015). Towards specifying pragmatic software reuse. ACM Int. Conf. Proc. Ser..

[9] Padhy N, Singh RP, Satapathy SC (2018). Software reusability metrics estimation: Algorithms, models and optimization techniques. Comput. Electr. Eng..

[10] Ghareb MI, Allen G (2021). Quality metrics measurement for hybrid systems (aspect oriented programming—object oriented programming). Tech. Roman. J. Appl. Sci. Technol..

[11] Thapar SS, Sarangal H (2020). Quantifying reusability of software components using hybrid fuzzy analytical hierarchy process (FAHP)-Metrics approach. Appl. Soft Comput. J..

[12] Heba A (2013). Review on aspect oriented programming. Int. J. Adv. Comput. Sci. Appl..

[13] Colyer A, Clement A (2005). Aspect-oriented programming with AspectJ. IBM Syst. J..

[14] Nerurkar NW, Kumar A, Shrivastava P (2010). Assessment of reusability in aspect-oriented systems using fuzzy logic. ACM SIGSOFT Softw. Eng. Notes.

[15] Olive S, Prof N, Mwangi W, Kimani S (2014). A metrics-based framework for measuring the reusability of object-oriented software components. J. Inf. Eng. Appl..

[16] Sahu K, Srivastava RK (2020). Needs and importance of reliability prediction: An industrial perspective. Inf. Sci. Lett..

[17] Frakes W, Terry C (1996). Software reuse: Metrics and models. ACM Comput. Surv..

[18] Singh PK, Sangwan OP, Singh AP, Pratap A (2015). A framework for assessing the software reusability using fuzzy logic approach for aspect oriented software. Int. J. Inf. Technol. Comput. Sci..

[19] Kumar R, Khan SA, Khan RA (2015). Durable security in software development: Needs and importance. CSI Commun..

[20] Chaudhary R, Chatterjee R (2013). Essence of reusability in aspect-oriented systems. ACM SIGSOFT Softw. Eng. Notes.

[21] Agrawal A (2020). Software security estimation using the hybrid fuzzy ANP-TOPSIS approach: Design tactics perspective. Symmetry Basel.

[22] Przybyłek A (2011). Systems evolution and software reuse in object-oriented programming and aspect-oriented programming. Lecture Notes Comput. Sci..

[23] Senthil, V. S. Quantitative assessment of inheritance hierarchies for aspect oriented software development using a proposed aspect inheritance reusability model. In *2019 International Conference on Automation, Computational and Technology Management, ICACTM 2019* (2019). 10.1109/ICACTM.2019.8776775.

[24] Sant’Anna, C., Garcia, A., Chavez, C., Lucena, C., & Von Staa, A. On the reuse and maintenance of aspect-oriented software: An assessment framework. In *Anais do XVII Simpósio Brasileiro de Engenharia de Software.* (pp. 15–30). SBC (2003, October).

[25] Kumar R, Alenezi M, Ansari MTJ, Gupta BK, Agrawal A, Khan RA (2020). Evaluating the impact of malware analysis techniques for securing web applications through a decision-making framework under fuzzy environment. Int. J. Intell. Eng. Syst..

[26] Abdulhameed OA, Yousuf AY, Abbas RH (2019). “Aspect oriented programming: Concepts, characteristics and implementation. Period. Eng. Nat. Sci..

[27] Kumar P (2012). Aspect-oriented software quality model: The AOSQ model. Adv. Comput. Int. J..

[28] Chidamber SR, Kemerer CF (1994). A metrics suite for object oriented design. IEEE Trans. Softw. Eng..

[29] Aggarwal KK, Singh Y, Kaur A, Malhotra R (2006). Empirical study of object-oriented metrics. J. Object Technol..

[30] Kumar R, Khan SA, Agrawal A, Khan RA (2018). Measuring the security attributes through fuzzy analytic hierarchy process: Durability perspective. ICIC Express Lett..

[31] Gordieiev O, Kharchenko V, Fominykh N, Sklyar V (2014). Evolution of software quality models in context of the standard ISO 25010. Adv. Intell. Syst. Comput..

[32] Suman A, Wadha M (2014). A comparative study of software quality models. Int. J. Comput. Sci. Inf. Technol..

[33] Côté MA, Suryn W, Georgiadou E (2007). In search for a widely applicable and accepted software quality model for software quality engineering. Softw. Qual. J..

[34] Doinea M, van Osch W (2010). Collaborative systems: Defining and measuring quality characteristics. J. Appl. Collabor. Syst..

[35] Al-Qutaish RE (2010). Quality models in software engineering literature: An analytical and comparative study. J. Am. Sci..

[36] Ampatzoglou A, Kritikos A, Kakarontzas G, Stamelos I (2011). An empirical investigation on the reusability of design patterns and software packages. J. Syst. Softw..

[37] Svahnberg M, Gorschek T (2017). A model for assessing and re-assessing the value of software reuse. J. Softw.: Evol. Process.

[38] Pacheco C, Garcia I, Calvo-Manzano JA, Arcilla M (2017). Reusing functional software requirements in small-sized software enterprises: A model oriented to the catalog of requirements. Requir. Eng..

[39] Bieman JM, Karunanithi S (1995). Measurement of language-supported reuse in object-oriented and object-based software. J. Syst. Softw..

[40] Duhan F, Bhatia PK (2022). Software reusability estimation based on dynamic metrics using soft computing techniques. Int. J. Comput..

[41] Zozas I, Ampatzoglou A, Bibi S, Chatzigeorgiou A, Avgeriou P, Stamelos I (2019). REI: An integrated measure for software reusability. J. Softw.: Evol. Process.

[42] Kumar R, Baz A, Alhakami H, Alhakami W, Agrawal A, Khan RA (2021). A hybrid fuzzy rule-based multi-criteria framework for sustainable-security assessment of web application. Ain Shams Eng. J..

[43] Arora K, Singhal A, Bansal A (2012). Correlation between various quality characteristics for aspect-oriented systems. Int. J. Comput. Appl..

[44] Kaur PJ, Kaushal S, Sangaiah AK, Piccialli F (2018). A framework for assessing reusability using package cohesion measure in aspect oriented systems. Int. J. Parallel Program.

[45] Kumar, P. & Singh, S. K. A comprehensive evaluation of aspect-oriented software quality (AOSQ) model using analytic hierarchy process (AHP) technique. In *Proceedings—2016 International Conference on Advances in Computing, Communication and Automation (Fall), ICACCA 2016* (2016). 10.1109/ICACCAF.2016.7748957.

[46] Santos A, Alves P, Figueiredo E, Ferrari F (2016). Avoiding code pitfalls in Aspect-Oriented Programming. Sci. Comput. Program.

[47] Sirbi K, Kulkarni PJ (2010). Metrics for aspect oriented programming-an empirical study. Int. J. Comput. Appl..

[48] Kumar, P. & Singh, S. K. A systematic assessment of aspect-oriented software development (AOSD) using JHotDraw application. In *Proceeding—IEEE International Conference on Computing, Communication and Automation, ICCCA 2016* (2017). 10.1109/CCAA.2016.7813840.

[49] Papamichail MD, Diamantopoulos T, Symeonidis AL (2019). Measuring the reusability of software components using static analysis metrics and reuse rate information. J. Syst. Softw..

[50] Zakaria, A. A. & Hosny, H. Metrics for aspect-oriented software design. In *Third International Workshop on Aspect Oriented Modeling* (2003).

[51] Rathee A, Chhabra JK (2022). Metrics for reusability of java language components. J. King Saud Univ. Comput. Inf. Sci..

[52] Upadhyay N, Despande BM, Agrawal VP (2011). Towards a software component quality model. Commun. Comput. Inf. Sci..

[53] Chaudhary, R. & Chatterjee, R. Reusability in AOSD—the aptness, assessment and analysis. In *ICROIT 2014—Proceedings of the 2014 International Conference on Reliability, Optimization and Information Technology* (2014) 10.1109/ICROIT.2014.6798291.

[54] Kaur, P. J. & Kaushal, S. Package level metrics for reusability in aspect oriented systems. In *2015 1st International Conference on Futuristic Trends in Computational Analysis and Knowledge Management, ABLAZE 2015* (2015). 10.1109/ABLAZE.2015.7155021.

